# Production of *Alternaria* Toxins in Yellow Peach (*Amygdalus persica*) upon Artificial Inoculation with *Alternaria alternate*

**DOI:** 10.3390/toxins13090656

**Published:** 2021-09-15

**Authors:** Jiajia Meng, Wenbo Guo, Zhihui Zhao, Zhiqi Zhang, Dongxia Nie, Emmanuel K. Tangni, Zheng Han

**Affiliations:** 1 Institute for Agro-Food Standards and Testing Technology, Shanghai Key Laboratory of Protected Horticultural Technology, Shanghai Academy of Agricultural Sciences, Shanghai 201403, China; mengjiajia@saas.sh.cn (J.M.); guowenbo@saas.sh.cn (W.G.); zhaozhihui@saas.sh.cn (Z.Z.); zhangzhiqi@saas.sh.cn (Z.Z.); niedongxia@saas.sh.cn (D.N.); 2 Organic Contaminants and Additives, Sciensano, 3080 Tervuren, Belgium; emmanuel.tangni@sciensano.be

**Keywords:** yellow peach (*Amygdalus persica*), *Alternaria* toxins, inoculation, production

## Abstract

The yellow peach (*Amygdalus persica*), an important fruit in China, is highly susceptible to infection by *Alternaria* sp., leading to potential health risks and economic losses. In the current study, firstly, yellow peaches were artificially inoculated with *Alternaria* *alternate*. Then, the fruits were stored at 4 °C and 28 °C to simulate the current storage conditions that consumers use, and the *Alternaria* toxins (ATs) contents from different parts of the fruits were analyzed via ultra-high-performance liquid chromatography-tandem mass spectrometry (UHPLC-MS/MS). The results showed that the growth of *A*. *alternate* and the ATs production were dramatically affected by the storage temperature. At 28 °C, the fungi grew rapidly and the lesion diameter reached about 4.0 cm within 15 days of inoculation, while, at 4 °C, the fungal growth was noticeably inhibited, with no significant change in the lesion diameter. To our surprise, high contents of ATs were produced under both storage conditions even though the fungal growth was suppressed. With an increase in the incubation time, the amounts of ATs showed a steady tendency to increase in most cases. Remarkably, alternariol monomethyl ether (AME), alternariol (AOH), and tenuazonic acid (TeA) were detected in the rotten tissue and also in the surrounding tissue, while a large amount of TeA could also be found in the healthy tissue. To the best of our knowledge, this is the first report regarding the production of ATs by the infection of *Alternaria* sp. in yellow peach fruits via artificial inoculation under regulated conditions, and, based on the evidence herein, it is recommended that ATs be included in monitoring and control programs of yellow peach management and food safety administration.

## 1. Introduction

Peach (*Prunus persica* L. Batsch), a climacteric stone fruit species, is the fourth most important fruit in China after orange, apple, and pear [1]. The yellow peach (*Amygdalus persica*), a kind of peach with yellow flesh, is favored by consumers because of its delicacy, nutrition, and unique function [2]. Unfortunately, this fruit is highly susceptible to fungal contamination, especially *Alternaria* sp., due to its high moisture content, high level of nutrients, thin skin, and potentially improper harvest or storage conditions, leading to crucial economic and material losses to the food industry and growers [3,4,5,6,7].

**Figure 1:**
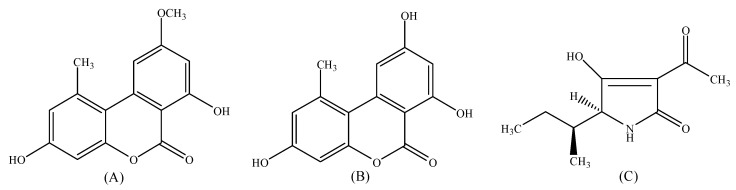
Chemical structures of alternariol monomethyl ether, AME (**A**); alternariol, AOH (**B**); and tenuazonic acid, TeA (**C**).

*Alternaria*, a common genus of ascomycete fungi including both plant-pathogenic and saprophytic species, is widespread in nature [8,9]. It can easily infect a large variety of crops in the fields and cause the spoilage of various fruits, grains, and vegetables [10,11]. *Alternaria* species are known to produce toxic secondary metabolites, and more than 70 *Alternaria* toxins (ATs) have been characterized so far. Among these metabolites, alternariol monomethyl ether (AME), alternariol (AOH), and tenuazonic acid (TeA) (Figure 1) are the ones most frequently found in a broad spectrum of fruits and processed fruit products, including apples, apple juice, tomatoes, tomato ketchup, citrus juice, grapes, peaches, etc. [12,13,14,15]. It has been reported that ATs have shown various toxicities and pose a serious health risk to humans and animals. ATs might be one of the most important factors for human esophageal cancer in Linxian, China [16]. TeA, with the highest toxicity among the *Alternaria* toxins, is toxic to many animal species, such as guinea pigs, rabbits, mice, dogs, and chickens [8,17]. AOH and AME are mutagenic and genotoxic and exhibit significant cytotoxicity in cell culture [18,19]. Due to their widespread occurrence and high toxicity, the European Food Safety Authority (EFSA) has established the threshold of toxicological concern (TTC) values as 2.5 ng kg^−1^ body weight per day for AOH and AME and 1500 ng kg^−1^ body weight per day for TeA [18]. The exposure levels of AOH, AME, and TeA reported in Belgium (12.6, 0.96, 104.3 ng kg^−1^ body weight per day) [20], China (2.4, 41.6, 6858 ng kg^−1^ body weight per day) [21], and Europe (71.6, 38.8, 1614 ng kg^−1^ body weight per day) [22] have confirmed that the TTC values for the *Alternaria* toxins are frequently exceeded, highlighting the necessity of thorough investigations regarding the production mechanisms of these toxins so as to establish efficient prevention and control strategies.

The growth of *Alternaria* sp. and the production of ATs are affected by multiple environmental factors, such as substrate, air humidity and temperature, of which temperature is the most important [23]. It has been suggested that 22–30 °C is the optimum growth temperature for *Alternaria* sp., whereas the fungi could also grow at a low temperature [24,25]. It was reported that the production of TeA at 28 °C in soybean-based media was higher than that at 4 °C on the same day upon inoculation within 10 d, while the production of AOH and AME was more favorable at 4 °C [26]. Therefore, *Alternaria* sp. has been demonstrated to be one of the key pathogens causing the spoilage of fruits stored at a low temperature due to the pathogen’s strong survivability under stress conditions. In Shanghai, China, the yellow peach is usually harvested in the summer and is stored at room temperature (about 28 °C) or in the refrigerator at 4 °C to retain freshness [25]. However, little is known about the infection of *Alternaria* sp. in regard to the production of ATs and spoilage in the yellow peach under storage conditions.

On the other hand, consumers are used to eating healthy, fresh yellow peaches, but, when fruits become slight decayed or develop small wounds, some consumers would remove the rotten parts and eat the intact portions. The major problem is whether the intact portions of yellow peaches that are infected by *Alternaria* sp. are really healthy. In other words, after infection with *Alternaria* sp., are the parts of the fruits without visible symptoms contaminated with ATs? Robiglio and López reported that AOH and AME could be detected not only in rotten fruits but also in asymptomatic sound tissues in apples [27]. However, to the best of our knowledge, no information has been found to fill the knowledge gap for this aspect of the yellow peach.

Herein, the aim of the present study was (i) to evaluate the fungal growth and production of AOH, AME, and TeA in yellow peaches upon artificial inoculation with *Alternaria alternate* stored at different temperatures and (ii) to investigate the distributions of these three ATs in different parts of the yellow peach to provide novel insights into the monitoring and control of the quality and safety of yellow peaches.

## 2. Results

### 2.1. Performance of UHPLC-MS/MS Method

To ensure the accuracy and reliability of the UHPLC-MS/MS method for the quantification of ATs, the established approach was carefully validated by the determination of linearity, sensitivity, recovery, precision, and matrix effect according to the recommendations of the European Commission Decision 2006/401/EC [28]. The linearity of the ATs is shown in Table 1. Good linear relationships with correlation coefficients (R^2^) > 0.99 were obtained. The limit of detection (LOD) and limit of quantification (LOQ) values were in the ranges of 0.07–0.11 µg kg^−1^ and 0.18–0.28 µg kg^−1^, respectively. The recoveries and intra- and inter-day precision values for ATs at the three fortified levels (10, 50, and 100 µg kg^−1^) are listed in Table 2. The mean recovery values ranged from 77.5% to 104.9%. The intra- and inter-day precision values were between 0.7% and 5.7% and 2.2% and 11.2%.

### 2.2. The Visual Appearance of Yellow Peaches Decay upon Inoculation with A. alternate

**Figure 2:**
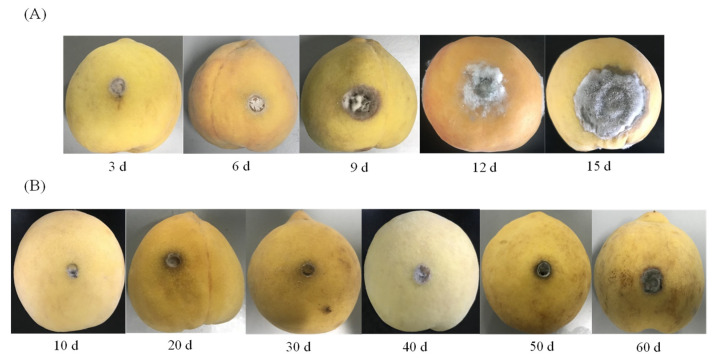
The symptoms of yellow peaches stored at 28 °C (**A**) and 4 °C (**B**) upon inoculation with *A. alternate*, respectively.

**Figure 3:**
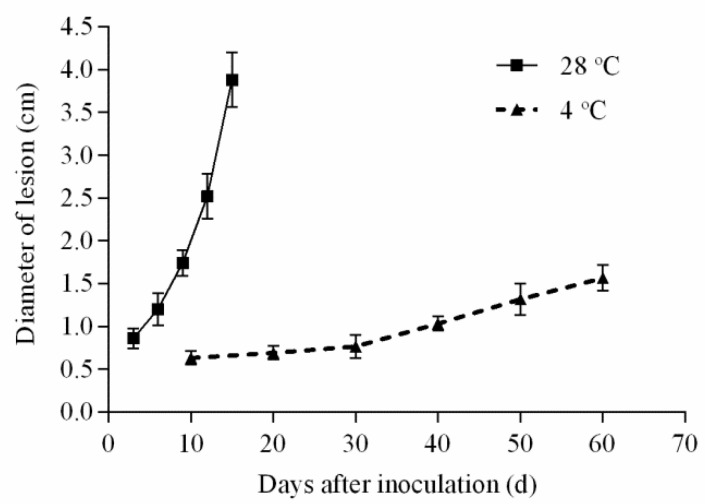
The lesion diameters on the surface of yellow peaches inoculated with *A. alternate* and stored at 28 °C and 4 °C, respectively.

After inoculation with *A. alternate*, the yellow peaches were stored at 28 °C for 15 d and 4 °C for 60 d, respectively, and the diameters of the lesions were determined. Significant differences were observed in the decay of yellow peach fruits under different storage temperatures (Figure 2 and Figure 3). At 28 °C, the fungal growth was relatively slow at the early stage (i.e., 3–6 d), but it was rapidly extended after 12 d. The lesions of the yellow peaches increased rapidly and extensive rot on the surface was observed 15 d post-inoculation. At 4 °C, the fungal growth was rather gradual before 30 d post-inoculation, and the growth rate was only 0.01 cm d^−1^. After 30 d, the growth rate increased slightly, but only to 0.03 cm d^−1^. Even on day 60, the diameter of the lesion was only 1.5 cm. In contrast with 28 °C, the fungus growth was inhibited at 4 °C; the rotten symptoms exhibited a slight and slow increase over the whole trial.

### 2.3. Production of Alternaria Toxins upon Inoculation with A. alternate

**Figure 4:**
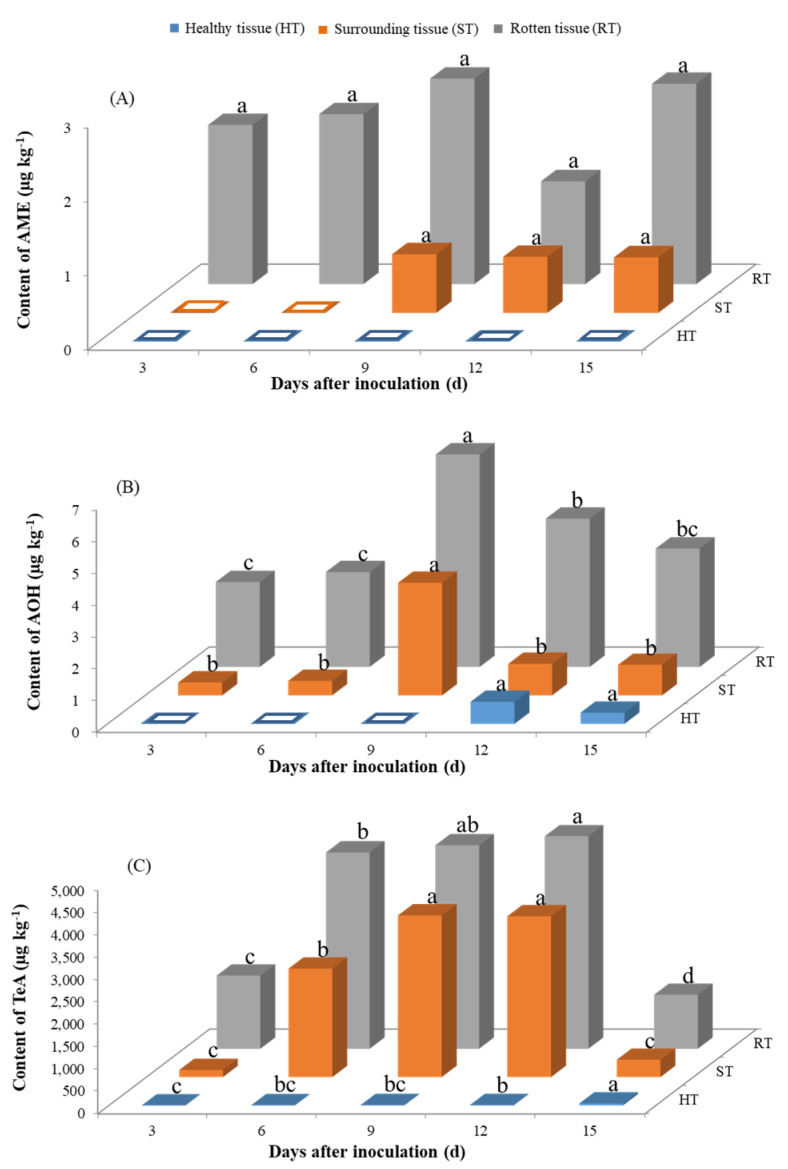
Contents of AME (**A**), AOH (**B**), and TeA (**C**) in yellow peaches stored at 28 °C upon inoculation with *A. alternate*. Note: For each toxin in the same part of the yellow peach, the small letters of a, b, c and d and their combinations were utilized to investigate the differences in the toxin contents with different incubation times. Different letters indicate the significance at *p* ≤ 0.05, while combinations and individual letters—e.g., ab and a—indicate no significant differences. Hollow squares indicate that the amount of ATs was lower than the LOQ.

**Figure 5:**
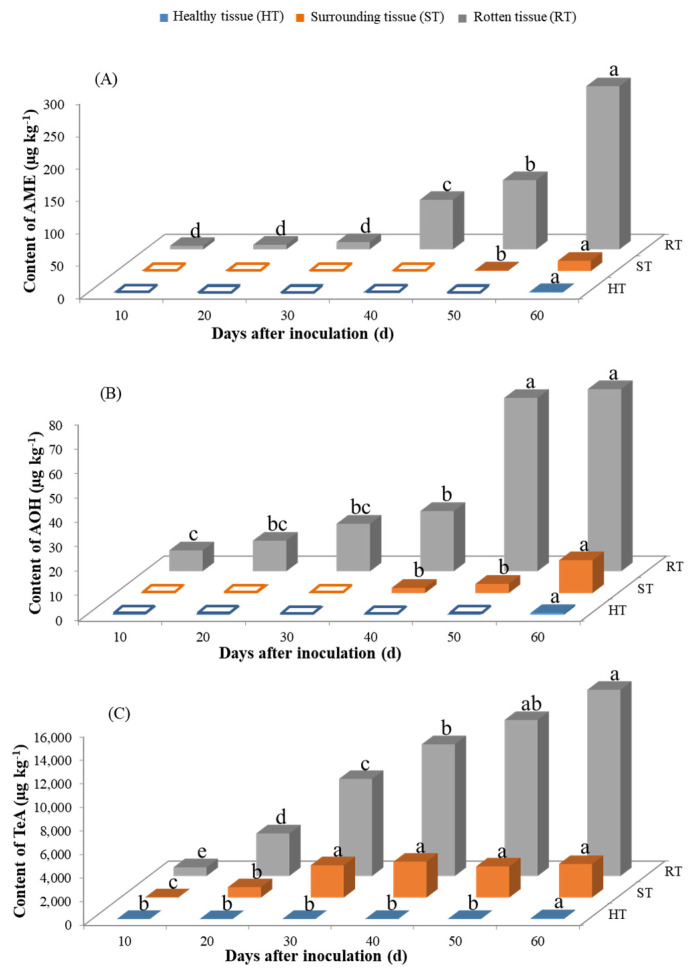
Contents of AME (**A**), AOH (**B**), and TeA (**C**) in yellow peaches stored at 4 °C upon inoculation with *A. alternate*. Note: For each toxin in the same part of the yellow peach, the small letters of a, b, c, d, and e and their combinations were utilized to investigate the differences in the toxin contents with different incubation times. Different letters indicate the significance at *p* ≤ 0.05, while combinations and individual letters—e.g., ab and a—indicate no significant difference. Hollow squares indicate that the amount of the ATs was lower than the LOQ.

The contents of ATs, including AME, AOH, and TeA, in yellow peaches stored at 28 °C and 4 °C were determined by UHPLC-MS/MS (Figure 4 and Figure 5). Overall, the ATs concentrations showed a steady increase in most cases under the two different storage conditions, with TeA being the most extensively produced toxin. The productions of AME and AOH were more favorable at 4 °C, while 28 °C seemed more suitable for the TeA production with the same incubation time—i.e., the contents were 4556.8 µg kg^−1^ at 28 °C and 743.5 µg kg^−1^ at 4 °C for TeA at around 9–10 d after inoculation, respectively.

#### 2.3.1. Production of *Alternaria* Toxins Stored at 28 °C upon Inoculation with *A. alternate*

The productions of AOH (up to 2.8 µg kg^−1^) and AME (up to 6.7 µg kg^−1^) were significantly lower than those of TeA (up to 4760.0 µg kg^−1^). AME was not detected in any healthy tissue (HT), and its amount in surrounding tissue (ST) was very low (no more than 0.8 µg kg^−1^). Relatively higher AME contents in the range of 1.4–2.8 µg kg^−1^ were found in rotten tissue (RT). Similarly, the amounts of AOH in RT and ST were in the range of 0.4–6.7 µg kg^−1^, and trace amounts of 0.7 and 0.4 µg kg^−1^ were found in HT at 12 and 15 d after inoculation. Dramatically higher amounts of TeA were produced by *A. alternate*, and the highest amounts of 3620.6 and 4760.0 µg kg^−1^ in ST and RT were achieved at 9 and 12 d after inoculation, respectively. The content of TeA in HT increased gradually and reached 43.8 µg kg^−1^ 15 d after inoculation.

#### 2.3.2. Production of *Alternaria* Toxins Stored at 4 °C upon Inoculation with *A. alternate*

Under this condition, the contents of AME, AOH, and TeA in RT gradually increased with the increase in inoculation time and reached maximums of 251.3, 74.2, and 15,819.2 µg kg^−1^ 60 d after inoculation, respectively. AME, AOH, and TeA could also be detected in ST, with the maximal contents being 15.7, 13.4, and 3043.5 µg kg^−1^, respectively. In HT, both AME and AOH could only be detected at day 60 at low concentration levels of 0.9 and 0.7 µg kg^−1^, respectively, while, for TeA, much higher amounts of up to 60.2 µg kg^−1^ were found at day 60.

## 3. Discussion

In Shanghai, the cultivation area of yellow peaches has reached 2700 hm^2^ in recent years, accounting for 50% of the total area of peaches. To ensure the safety and quality of this fruit and minimize economic losses, this study thoroughly investigated the infection of *Alternaria* sp. and assessed the presence of related toxins during storage.

A reliable and accurate UHPLC-MS/MS method based on the QuEChERS technique was developed for the simultaneous determination of typical ATs, including AME, AOH, and TeA. After careful validation through the determination of linearity, sensitivity, recovery, and precision, the analytical performance of the established approach was found to be comparable or even better than that of the previous methods [10,29], and it was evidently suitable to be applied to evaluate the contents of ATs in yellow peaches.

Environmental factors, especially temperature, played an important role in the fungal growth and ATs production [23]. The optimum temperature for the growth of *Alternaria* sp. was 22–30 °C, while the fungus could also grow at low temperatures, even at −5 °C [24,25]. To simulate the real-life situation, 4 °C (the typical household temperature in the refrigerator) and 28 °C (room temperature) were chosen as the storage conditions in this study. The temperature of 28 °C was suitable for fungal growth and the lesion diameter reached almost 4.0 cm within 15 d, which was similar to the results of fungal growth in soybean medium [26] or tomato medium [30] inoculated with *A. alternata*. At 4 °C, the fungal growth was inhibited (the growth rate was about 0.01–0.03 cm d^−1^) and the decay of the yellow peach was slight even though the storage time was extended to 60 d (the lesion diameter was only about 1.5 cm). These results were consistent with those obtained by Vaquera et al. [31] in which the growth of *Alternaria* sp. was also inhibited at a low temperature (6 °C) and the growth rate was only 0.52 mm d^−1^ on tomato medium. Similar results were also obtained in strawberries with the maximum storage time (1–5 weeks) at 6 °C, while there was a maximum of 8 d for those samples stored at 22 °C in relation to the fungal growth [25]. 

Besides causing the fruits to rot, the fungi could also produce a variety of ATs. In this work, three typical toxins, AME, AOH, and TeA, were found in yellow peach fruits stored at 4 °C and 28 °C, which was consistent with those produced in other fruits and vegetables—i.e., tomatoes [32], citrus [33], etc. Among these three toxins, the amount of TeA was about 100 orders of magnitude larger than that of AOH and AME. Similar results were also found by Cabral et al. [32] in a tomato-based medium; for example, the production of AME was 4.8 µg g^−1^, while the content of TeA was up to 363.6 µg g^−1^ under the same conditions. It was not surprising to find that TeA would be produced the most by *A. alternate,* since TeA has a rather different type compared to other *Alternaria* toxins, which was consistent with the results of previous studies [32,34].

The amounts of the three ATs exhibited a continuous increase along with the incubation time, similar to the results reported for wheat, rice, and tomato matrices [31,35]. In a few cases, the contents of ATs such as AOH and TeA decreased at a late storage stage, which might be due to the insufficient nutrients in the rotten yellow peaches, the accumulation of inhibitory metabolites, or the transformation of ATs into new compounds [36]. Low temperature was usually proposed as a good strategy for the post-harvest storage and preservation of peaches [37] because it could inhibit the fungal growth and prevent the decay of the fruits. However, in the present work, at the temperature of 4 °C, with the extension of the incubation time, the amount of ATs accumulated rapidly and could reach a high content, though the growth of fungi was suppressed.

To clarify the distributions of ATs in yellow peaches after the inoculation of *A. alternate*, we further analyzed the ATs contents in different parts of yellow peaches. As expected, large amounts of ATs were observed in rotten tissue (RT), but, surprisingly, the ATs were also detectable in surrounding tissue (ST) and even in healthy tissue (HT), especially for TeA, which was considered to be the most toxic of the ATs. Similar results were reported in apples by Robiglio and Lopez, in which AOH and AME were detected in asymptomatic tissues [27]. Jiang et al. [38] also found that ATs produced in infected tomatoes can rapidly spread from infected to healthy parts. It is worth emphasizing that, although the rotting area at 4 °C occupied just approximately 2% of the whole surface area of the peach, the TeA content in yellow peaches was extremely high, including in the healthy parts. As a consequence, significant potential health risks were related to the rotten yellow peaches, even with the consumption of only the healthy parts, so a call for thorough monitoring and control programs for ATs in fruits and their products is necessary.

To reduce health risks, based on our investigations, some suggestions were thus proposed: first, the fruits should be intact without any physical defect or surface damage, and they ought to be stored at a low temperature for a short time. Once the fruits have been infected with toxigenic fungi, yellow peaches should not be consumed—either the decayed or un-decayed parts—due to the potential contamination by *Alternaria* toxins. Moreover, considering a variety of metabolites produced by *Alternaria* sp., a higher number of analytes—i.e., altenuene (ALT) and altertoxin (ATX–I)—could be used in further studies.

## 4. Conclusions

In the present study, the infection of *Alternaria* sp. and the production of related toxins were thoroughly investigated in yellow peaches under common storage conditions (28 °C and 4 °C) for the first time. High amounts of ATs were produced by the infected fungi under both conditions, whether the fungal growth was promoted at 28 °C or inhibited at 4 °C. As expected, after inoculation, AOH, AME, and TeA were detected in rotten tissue and also the surrounding tissue, but, worse than that, high amounts of TeA and trace amounts of the others could be found even in the healthy tissue. These interesting observations provide insight in regard to the quality and safety control of yellow peaches, and the findings also provide direct evidence for the development of the efficient management of various fruits.

## 5. Materials and Methods

### 5.1. Reagents and Chemicals

Acetonitrile and methanol (HPLC grade) were obtained from Merck (Darmstadt, Germany). Sodium chloride (NaCl, analytical grade), anhydrous magnesium sulfate (MgSO_4_, analytical grade) and ammonium acetate (HPLC grade) were supplied by ANPEL (Shanghai, China). Purified water was prepared using a Milli-Q Plus apparatus (Millipore, Billerica, MA, USA).

The analytical standards (stock solutions) of AOH (100.0 µg mL^−1^), AME (100.3 µg mL^−1^) and TeA (101.1 µg mL^−1^) dissolved in acetonitrile were purchased from Romer labs (Union, MO, USA).

Yellow peaches with uniform fruit size, ripeness and the absence of physical defects or apparent infections were collected from local retail markets and brought to the laboratory in pre-sterilized polyethylene bags.

### 5.2. Fungal Material and the Preparation of Spore Suspension

The strain of *A. alternate* (GenBank accession number MW517324) was isolated from the yellow peaches by single spore isolation. It was stored both on PDA slants at 40 °C and in 40% glycerol at −40 °C in the Herbarium of the Institute of Agro-food Standard and Testing Technology, Shanghai Academy of Agricultural Sciences, Shanghai, China.

After activation, the fungus was inoculated on PDA plates and then incubated at 28 °C for 7 d. Conidia were collected with a coating stick within sterile water and filtered through Miracloth. Spore suspension was adjusted to a concentration of 2 × 10^6^ conidia mL^−1^ using a Thoma counting chamber and used for inoculation.

### 5.3. Artificial Inoculation

Before inoculation, the yellow peaches were firstly washed with tap water and submerged in 1% sodium hypochlorite solution for 2 min, and then they were rinsed with sterile distilled water and dried with sterile filter paper. Under aseptic conditions, the yellow peaches were three-times injured on the equatorial section with the help of a sterilized borer. Then, 20 µL of spore suspension was inserted into the wound through a sterilized micropipette, and the fruits that were inserted with 20 µL sterile water were used as the control. All of the inoculated yellow peaches were stored at 28 °C and 4 °C in covered plastic trays lined with filter paper moisturized with sterile water.

**Figure 6:**
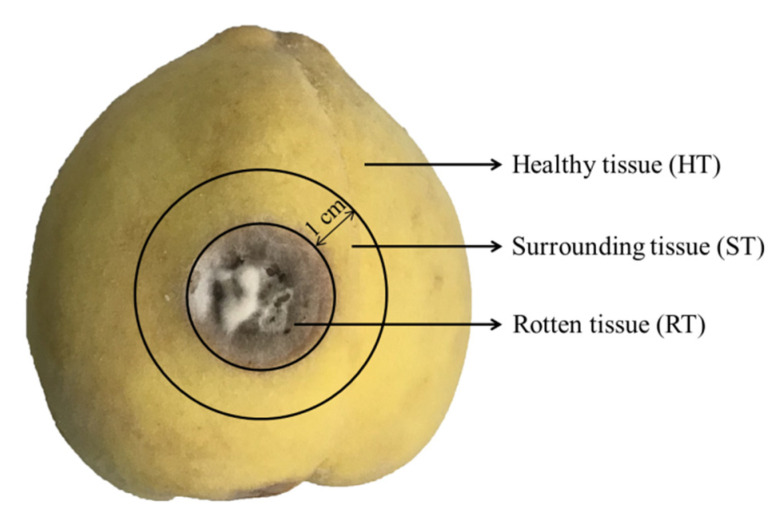
Schematic illustration of different parts of yellow peaches for *Alternaria* toxins (ATs) analysis.

At specific time intervals after inoculation, five peaches of each treatment were taken to the laboratory, where the diameters of the lesions were assessed. The samples, including RT, ST, and HT (as shown in Figure 6), were separately collected and homogenized to be used for the analysis of ATs by UHPLC-MS/MS.

### 5.4. UHPLC-MS/MS Analysis

The UHPLC analysis was performed via a Waters ACQUITY Ultra High-Performance LC system (Waters, Milford, MA, USA). Separation was achieved on an XBridge^®^ BEH C_18_ column (3.0 mm × 100 mm, 2.5 µm) at 40 °C with a flow rate of 0.4 mL min^−1^. The mobile phase consisted of methanol (A) and water containing 5 mmol L^−1^ ammonium acetate (B). A linear gradient elution program was set as follows: initial 10% A; 1 min, 10% A; 5 min, 90% A; 6 min, 90% A; 6.5 min, 10% A; 8 min, 10% A. The injection volume was 3 µL.

For the MS/MS analysis, a Waters TQS mass spectrometer system (Waters, Milford, MA, USA) was used in positive electrospray ionization mode (ESI^+^) with the following parameters: interface voltages of capillary, 2.5 kV; source temperature, 150 °C; desolvation temperature, 500 °C. The nebulizing gas and desolvation gas flow rates were 7.0 bar and 1000 L/h, respectively. Multiple reaction monitoring (MRM) mode was used for the quantification and confirmation of the ATs with the parameters shown in Table 3.

### 5.5. Sample Preparation

The ATs determination was carried out according to the method previously established in our lab [29], with minor modifications. Briefly, the homogenized samples (2.0 g) were put into a 50 mL centrifuge tube and 10 mL of acetonitrile containing 1% formic acid was added. The mixture was vortexed for 30 s, and ultrasonic extraction took 40 min. Subsequently, 0.5 g of anhydrous magnesium sulfate and 0.5 g of sodium chloride were added to the slurry and vigorously shaken for 30 s immediately. After centrifugation at 4500 rpm for 10 min, 5 mL of the supernatant was transferred into 10 mL centrifuge tubes and dried by nitrogen stream at 40 °C. The residues were re-dissolved in 1 mL acetonitrile/water containing 5 mmol L^−1^ ammonium acetate (20/80, *v*/*v*), passed through a 0.22 µm PTFE membrane filter to be ready for analysis. Once the concentrations of ATs in the samples were out of the linear range, they were appropriately diluted to fit the calibration curve and re-analyzed.

### 5.6. Method Validation

The standards of the ATs were spiked into acetonitrile/water containing 5 mmol L^−^^1^ ammonium acetate (20/80, *v*/*v*) solutions and blank matrix, respectively, to yield a concentration sequence of 0.1, 0.2, 0.3, 0.5, 1, 5, 10, 50, 100 and 200 ng mL^−1^. The calibration curves were created by plotting the responses versus the concentrations. The matrix effect was assessed by the ratio of the slope matrix to slope solvent, where the slope matrix and slope solvent indicated the slope of the matrix-matched calibration curve and standard calibration curve, respectively. The sensitivity was determined by the limit of detection (LOD) and limit of quantification (LOQ), which were expressed as the concentrations of the toxins that produced signal-to-noise ratios (S/N) of 3 and 10 in the matrix, respectively. Recovery intra- and inter-day precision tests were performed in quintuplicate using blank samples spiked with low, intermediate and high concentration levels (10, 50 and 100 µg kg^−1^) of ATs. The recovery was calculated by comparing the measured concentration using the matrix-matched calibration curves with the spiked (theoretical) concentration of each toxin. The intra- and inter-day precisions were evaluated by the relative standard deviations (RSDs) determined on the same day and the values for five consecutive days.

### 5.7. Statistical Analysis

All of the tests were performed with three replications, and the results were represented by their mean values and the standard deviations. The statistical analysis of the data was performed by analysis of variance (one-way ANOVA). The differences between the treatments were evaluated by the Tukey method (*p* ≤ 0.05).

## Figures and Tables

**Table 1:** Linearity, sensitivity, and matrix effect of different ATs.

*Alternaria* Toxins	Linear Range	Linear Equation	Correlation Coefficient (R^2^)	LOD (µg kg^−1^)	LOQ (µg kg^−1^)	Matrix Effects
AME	0.3–200	y = 2662.59x + 718.912	0.998	0.07	0.18	74.65
AOH	0.3–200	y = 3335.09x + 540.209	0.999	0.11	0.28	110.91
TeA	0.2–200	y = 1082.09x + 67.7107	0.998	0.07	0.20	41.05

**Table 2:** Recovery and intra- and inter-day precision values for ATs in yellow peaches.

*Alternaria* Toxins	Spiked Levels (µg kg^−1^)	Recovery (Mean ± SD, %)	Intra-Day Precision (RSD, %)	Inter-Day Precision (RSD, %)
AME	10	104.9 ± 1.2	5.5	6.0
	50	96.3 ± 7.6	5.6	4.1
	100	98.6 ± 6.2	4.1	3.3
AOH	10	96.0 ± 7.4	5.7	9.7
	50	91.1 ± 6.0	3.1	8.1
	100	97.8 ± 3.5	1.9	6.0
TeA	10	77.5 ± 0.9	1.8	11.2
	50	97.3 ± 4.7	2.7	4.7
	100	85.0 ± 7.0	0.7	2.2

**Table 3:** MS/MS parameters for the determination of ATs.

*Alternaria* Toxins	Precursor Ions (*m*/*z*)	Product Ions (*m*/*z*)	Dwell Times (s)	Cone Voltage (V)	Collision Energy (eV)
AME	273.0 [M + H]^+^	128.1/258.0 *	0.018	54/54	26/25
AOH	259.0 [M + H]^+^	185.1 ^*^/213.1	0.018	64/64	28/24
TeA	198.1 [M + H]^+^	125.0/153.1 *	0.018	42/42	16/12

* Primary production.
